# The deafness gene DFNA5 induces programmed cell death through mitochondria and MAPK-related pathways

**DOI:** 10.3389/fncel.2015.00231

**Published:** 2015-07-16

**Authors:** Sofie Van Rossom, Ken Op de Beeck, Vesna Hristovska, Joris Winderickx, Guy Van Camp

**Affiliations:** ^1^Department of Biomedical Sciences, Center of Medical Genetics, University of AntwerpAntwerp, Belgium; ^2^Functional Biology, Department of BiologyKU Leuven, Heverlee, Belgium

**Keywords:** hearing loss, programmed cell death, oxidative stress, mitochondria, MAPK, DFNA5

## Abstract

Cell death exists in many different forms. Some are accidental, but most of them have some kind of regulation and are called programmed cell death. Programmed cell death (PCD) is a very diverse and complex mechanism and must be tightly regulated. This study investigated PCD induced by *DFNA5*, a gene responsible for autosomal dominant hearing loss (HL) and a tumor suppressor gene (TSG) involved in frequent forms of cancer. Mutations in *DFNA5* lead to exon 8 skipping and result in HL in several families. Expression of mutant DFNA5, a cDNA construct where exon 8 is deleted, was linked to PCD both in human cell lines and in *Saccharomyces cerevisiae*. To further investigate the cell death mechanism induced by mutant DFNA5, we performed a microarray study in both models. We used wild-type *DFNA5*, which does not induce cell death, as a reference. Our data showed that the yeast pathways related to mitochondrial ATP-coupled electron transport chain, oxidative phosphorylation and energy metabolism were up-regulated, while in human cell lines, MAP kinase-related activity was up-regulated. Inhibition of this pathway was able to partially attenuate the resulting cell death induced by mutant DFNA5 in human cell lines. In yeast, the association with mitochondria was demonstrated by up-regulation of several cytochrome c oxidase (COX) genes involved in the cellular oxidative stress production. Both models show a down-regulation of protein sorting- and folding-related mechanisms suggesting an additional role for the endoplasmic reticulum (ER). The exact relationship between ER and mitochondria in DFNA5-induced cell death remains unknown at this moment, but these results suggest a potential link between the two.

## Introduction

Cell death is a fundamental process of all organisms and inherent to life. The role of programmed cell death (PCD) in the pathology of hearing loss (HL) has been well studied and seems to play a prominent role, especially in the development of the vertebrate inner ear and in the morphogenesis of the semicircular canals (Fekete et al., 1997; Nishikori et al., 1999; Leon et al., 2004). Several genes related to PCD-induced HL have been identified and many of these are related to the mitochondria (Estivill et al., 1998; Jacobs et al., 2005; Ding et al., 2013). Mitochondria are key players during PCD and dysfunction of the mitochondria has been linked to the pathogenesis of many diseases, including HL. Mitochondria are the main producers of cellular ATP due to oxidative phosphorylation. However, this process also generates reactive oxygen species (ROS) (Raha and Robinson, 2000). Due to the mitochondrial production of ROS, the reduced DNA repair capacity and the close proximity of mtDNA to ROS generation sites, mtDNA (mitochondrial DNA) is very susceptible to mutations. Enhanced oxidative stress will have detrimental effects on cellular health and plays a major role during aging, mutagenesis, and cell death (Gredilla and Barja, 2005; Maynard et al., 2009; Gredilla et al., 2010).

Due to the relative slow speech perception detoriation, it is assumed that DFNA5-related HL is due to cochlear dysfunction. The cochlea seems to be highly susceptible to the detrimental effects of mitochondrial damage due to the post-mitotic character of its sensory epithelium (Sha et al., 2001).

It is estimated that in the Caucasian population at least 5% of the post-lingual non-syndromic HL is due to mutations in the mtDNA (Estivill et al., 1998; Jacobs et al., 2005). Most mutations in mtDNA affect the mitochondrial *MTRNR1* and the *MTTS1* genes encoding respectively a mitochondrial 12S rRNA and a tRNA^Ser^. Mutations in these mitochondrial genes lead to variable clinical severity of HL due to impaired mitochondrial tRNA metabolism and protein synthesis (Casano et al., 1999; Fischel-Ghodsian, 1999; Jin et al., 2007; Ding et al., 2013; Dowlati et al., 2013). Also many of the nuclear DNA mutations leading to HL are related to mitochondrial dysfunction and PCD. These include genes such as *OPA1, TIMM8A, SMAC/DIABLO, MPV17, PDSS1, BCS1L, SUCLA2, C10ORF2, COX10, PLOG1*, and *RRM2B* (Roesch et al., 2002; Antonicka et al., 2003; Payne et al., 2004; Mollet et al., 2007; Cheng et al., 2011; Meyer Zum Gottesberge et al., 2012; Luo et al., 2013). These genes contribute to various fundamental mitochondrial aspects, such as mitochondrial protein transport, mitochondrial fragmentation, and oxidative phosphorylation. Mutations in these genes are thought to induce mitochondrial stress and trigger cell death in the cochlea leading to HL. The explanation for the tissue specific effect of these genes leading to more prominent cell death in the cochlea and the hair cells remains unknown at this moment.

In addition to the genes directly associated with the mitochondria, two other genes related to apoptosis, a specific form of PCD, have been linked with hearing loss. *MSBR3*, a gene encoding a methionine sulfoxide reductase, is associated with caspase-3 activity. This can initiate apoptosis, eventually leading to degeneration of the inner hair cells and recessive non-syndromic HL (Ahmed et al., 2011). Overexpression of the *TJP2* gene, due to a genomic duplication, altered the expression of several apoptotic genes, inducing a dominant non-syndromic form of HL (Walsh et al., 2010).

From these examples, it is clear that a prominent association exists between (mitochondria-related) PCD and non-syndromic HL. Many forms of HL, such as age-related hearing impairment (ARHI), noise-induced hearing loss (NIHL), monogenic forms of HL and ototoxicity, have been associated with dysfunctional mitochondria and PCD, underscoring the importance and the need to further investigate the role of PCD in the pathogenesis of deafness (Casano et al., 1999; Sha et al., 2009; Someya et al., 2009; Liu et al., 2010; Chen et al., 2012).

One of the monogenic deafness genes that is related to PCD is *DFNA5*. *DFNA5* was originally identified as a gene responsible for an autosomal dominant form of HL in a Dutch family (Van Laer et al., 1998). Today, eight families with mutations in *DFNA5* (mut*DFNA5*) associated with HL have been identified. The phenotype of the HL is very similar with the exception of the age-of-onset which varies from 15 to 50 years old. The HL is symmetric and starts in the high frequencies, but spreads to the lower frequencies in a later stage. Four of the eight mutations differ at the genomic level, but they all result in skipping of exon 8, yielding an immature truncated protein (Yu et al., 2003; Bischoff et al., 2004; Cheng et al., 2007; Park et al., 2010; Nishio et al., 2014). The structure of DFNA5 is unknown at this moment, but hydrophobic cluster analysis revealed that DFNA5 consists of two regions, domain A and domain B, connected by a hinge region (Op de Beeck et al., 2011). Mutations in *DFNA5* result in a truncated protein lacking the last part of domain B due to a premature stop codon. This premature stop codon is caused by skipping of exon 8 as a result of the different genomic mutations in DFNA5 present in the families. Op de Beeck et al. (2011) have also demonstrated that only the first part, domain A, present in both wild-type and mutDFNA5, is sufficient to induce cell death after transfection in human cell lines. Domain B did not have any cell death inducing capacity. This led to the hypothesis that domain B will normally shield this cell death-inducing domain A to avoid inappropriate activation of domain A. Due to the partial lack of domain B in mutDFNA5, this shielding may not be possible, leading to a constitutive activation of mutDFNA5 (Op de Beeck et al., 2011).

In addition to HL, wild-type *DFNA5* (wt*DFNA5*) has also been correlated with several forms of cancer, such as breast, colorectal, hepatocellular, and gastric cancer. Endogenous *DFNA5* is epigenetically silenced by hypermethylation in cancer cells resulting in a decreased *DFNA5* expression level. Based on these findings, it is hypothesized that *DFNA5* is a tumor suppressor gene (TSG) (Akino et al., 2007; Kim et al., 2008a,b; Wang et al., 2013).

The function of DFNA5 remained unknown for a long time, but previous functional studies by Op de Beeck et al. (2011) revealed that DFNA5 induces a growth defect in mut*DFNA5*-transfected HEK293T cells, as well as other cells, leading to PCD (Op de Beeck et al., 2011). The cell death-inducing capacity of DFNA5 was not only restricted to human cell lines, but was also observed in the yeast model *Saccharomyces cerevisiae* (Van Rossom et al., 2012). This inspired us to use these two different model organisms to further elucidate the mechanisms related to *DFNA5*.

The *Saccharomyces cerevisiae* yeast model has several advantages as a model organism compared to human cell lines. The rapid growth, inexpensive media and the relatively easy genetic modifications, made the budding yeast *Saccharomyces cerevisiae* a valuable model to unravel regulators of different human pathologies (reviewed in Winderickx et al., 2008). Thirty percent of known genes involved in human diseases have an ortholog in yeast and due to this high degree of conservation, yeast is very suitable for fundamental research to identify core regulators of diverse signaling mechanisms (Foury, 1997). This was demonstrated in particular for mechanisms related to PCD where numerous yeast homologs of human genes related to cell death have been identified to play a common role (Wissing et al., 2004; Buttner et al., 2007; Madeo et al., 2009). The conservation of PCD mechanisms has been confirmed for DFNA5-related PCD in a previous study using the budding yeast *Saccharomyces cerevisiae* (Van Rossom et al., 2012). This study demonstrated the value of yeast to unravel PCD mechanisms-related to human genes. Transformation of mutant *DFNA5* (mut*DFNA5*) in yeast led to the induction of PCD. Several mitochondrial proteins, such as Fis1, Por1, Aac1 and Aac3, were shown to be involved. Moreover, mutDFNA5 was found to co-localize with a mitochondrial marker protein (Westermann and Neupert, 2000). These former observations established a role for mitochondria in DFNA5-related cell death in yeast and demonstrated the value of yeast as a model organism to unravel DFNA5-related (mitochondrial) HL.

In the current study, we performed a transcriptomic analysis and confirmed the significance of mitochondria in *DFNA5*-induced cell death in *Saccharomyces cerevisiae*. Additionally, Gene Ontology (GO) analysis suggested a role for the endoplasmic reticulum (ER). The latter observation was not only present in *Saccharomyces cerevisiae*, but was also confirmed in human cell lines. Furthermore, we show that the MAPK pathways, namely the induction of the extracellular signal regulated kinase (ERK) and c-Jun N-terminal kinase (JNK), are activated upon mut*DFNA5* transfection in human cell lines. Our data suggest the presence of a cellular adaptive response related to mitochondria, MAPK pathways and potentially for the ER. The exact correlation between those processes and DFNA5 remains unclear but further study will lead to a better understanding of DFNA5-induced cell death mechanisms and to HL-related to mitochondrial damage in general.

## Material and methods

### Yeast

#### Yeast strains and growth conditions

In this study, we used the BY4741 (MATa his3Δ1 leu2Δ0 met15Δ0 ura3Δ0) wild-type strain (Brachmann et al., 1998). Full-length cDNA of either wt*DFNA5* or mut*DFNA5* was isolated and amplified as previously described (Gregan et al., 2003). Amplified products were ligated into yeast pYX212 plasmid containing an HA-marker (Clontech, Mountain View, CA, USA) using *EcoRI* and *BamHI* restriction sites. All constructs were verified by bidirectional sequencing on an ABI genetic analyser 3130 × l (AppliedBiosystems, FosterCity, CA, USA).

**Figure 1:**
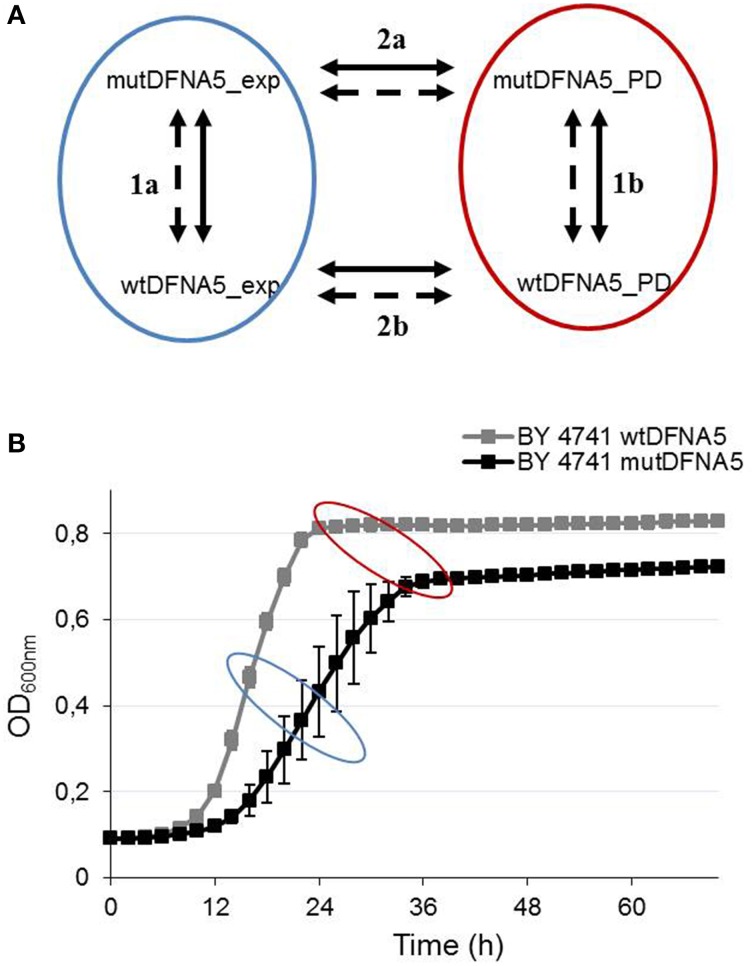
**Yeast microarray design. (A)** Illustration of the different comparisons that were made between the RNA samples. RNA was collected from yeast strains transformed with either wt*DFNA5* or mut*DFNA5* at two different time-points. The bold and dashed lines represent the color flip of each RNA sample. **(B)** Growth profile of the *Saccharomyces cerevisiae* BY4741 background strain transformed with either wt*DFNA5* (gray squares) or mut*DFNA5* (black squares). The blue and red ellipse illustrate respectively the mid-exponential and post-diauxic time-points when RNA was collected.

Yeast strains were grown at 30°C in selective medium containing 2% glucose (SD-URA). Fifty milliliter yeast cultures, transformed with either wt*DFNA5* or mut*DFNA5*, were harvested in mid-exponential phase (OD_600 nm_ = 3.5−4.2) and at the post-diauxic shift (OD_600 nm_ = 7.4−8.4) (Figure 1). Standard transformation techniques were applied for these transformations (Gietz et al., 1992).

#### RNA extraction yeast

RNA was collected from yeast at mid-exponential phase and just after the post-diauxic shift using RNA pure kit following the manufactures instructions (GenHunter® Corporation, Nashville, TN, USA). All steps were performed in duplo at 4°C to avoid RNA degradation. This resulted in eight different RNA samples: mid-exponential wt*DFNA5*, (WT_E1 and WT_E2), mid-exponential mut*DFNA5* (Mut_E1 and Mut_E2), post-diauxic wt*DFNA5* (WT_S1 and WT_S2), and post-diauxic mut*DFNA5* (Mut_S1 and Mut_S2) (Figure 1).

#### Microarray design and analysis

Microarray experiments were performed at the VIB Nucleomics Core (www.nucleomics.be). Before labeling, RNA concentration and purity were determined spectrophotometrically using the Nanodrop ND-1000 (Nanodrop Technologies). RNA integrity was assessed using a Bioanalyzer 2100 (Agilent). Per sample, an amount of 1 μg of total RNA spiked with 10 viral polyA transcript controls (Agilent, Santa Clara, CA, USA) was converted to double stranded cDNA in a reverse transcription reaction. Subsequently, the samples were converted to antisense cRNA, amplified and labeled with Cyanine 3-CTP (Cy3) or Cyanine 5-CTP (Cy5) in an *in vitro* transcription reaction according to the manufacturer's protocol (Agilent, Santa Clara, CA, USA). A mixture of purified and labeled cRNA (Cy3 label: 5 pmol; Cy5 label: 3.5 pmol) was hybridized on Agilent Yeastv2 arrays followed by washing, according to the manufacturer's procedures. To assess the raw probe signal intensities, arrays were scanned using the Agilent DNA MicroArray Scanner and probe signals were quantified using Agilent's Feature Extractor software (version 10.5.1.1).

In total four different comparisons were made. Each comparison was done in duplo by a color flip. The different comparisons are shown in Figure 1A. Gene expressions of strains transformed with wt*DFNA5* were compared to strains transformed with mut*DFNA5*, both in mid-exponential phase (comparison 1a) and at the post-diauxic shift (comparison 1b). Additionally, comparisons were made between the mid-exponential and the post-diauxic shift of either wt*DFNA5*-(comparison 2b) or mut*DFNA5*-(comparison 2a) transformed cells.

Analysis of the microarray data was performed using the R package LIMMA (http://www.bioconductor.org) (Gentleman et al., 2004). Fold changes were computed using raw Cy3 and Cy5 intensities values provided by Agilent's Feature Extractor software (version 10.5.1.1) and loess normalization and background correction were performed to determine the log2-ratios per array. Differential expression was assessed via the moderated t-statistic, described in (Smyth, 2004). To control the false discovery rate, multiple testing correction was performed. This generated four differentially expressed gene lists. All the (raw) data files have been completely uploaded to the gene expression omnibus and have been stored under accession number GSE70169.

### HEK293T cells

#### Cell culture and growth conditions

The microarray experiments in human cell lines were performed using Human Embryonic Kidney 293T cells (HEK293T). HEK293T cells were subcultured in 60 mm dishes at a density of 2 × 10^6^ cells in Dulbecco's modified Eagle's medium containing 4500 mg/L glucose supplemented with 10% (v/v) fetal calf serum, 100 U/ml penicillin, 100 μg/ml streptomycin, and 2 mM L-glutamine. Overnight cultures were transfected with either control (empty EGFP vector), wt*DFNA5* or mut*DFNA5* using lipofectamine. Six hours post transfection, cells were harvested using Triple-x reagent for RNA extraction. All products for human cell line cultures were obtained from Invitrogen (San Diego, CA, USA). Full-length cDNA of either wt*DFNA5* or mut*DFNA5* was isolated and amplified as previously described (Gregan et al., 2003).

#### RNA extraction

An RNeasy mini kit was used for RNA extraction from transfected HEK293T cells (Qiagen, Hilden, Germany) at different time-points. For the microarray experiment, RNA was extracted 12 h post-transfection. For the gene analysis by real time rtPCR, RNA was extracted at either 3, 6, 12, 15, 18, 20, or 24 h post-transfection. For the microarray experiment, the integrity of the resulting RNA was checked on an automated Experion electrophoresis system (Biorad, Hercules, CA, USA).

#### Microarray design and analysis

The “Totalprep RNA Amplification” kit was used to amplify the RNA samples (Illumina, Ambion, Austin, TX, USA). Doublestranded-cDNA was generated from the mRNA fractions followed by an *in vitro* transcription reaction which produced cRNA strands containing biotin-UTP nucleotides. Seven Hundred Fifty nano gram of the resulting cRNA samples were hybridized to an Illumina human HT12v3 beadchip (Illumina, San Diego, CA, USA).

Six independent biological replicates were used for either wt*DFNA5*- or mut*DFNA5*-transfected HEK293T cells and loaded on the chip. Overnight hybridization at 58°C was followed by washing and streptavidin-Cy3 dye labeling (Amersham, Buckinghamshire, England). An Illumina Iscan equipped with Iscan control software was used to measure the intensity values and XY coordinates for every probe on the array. The resulting data files were then analyzed using the R package “Beadarray v1.14.0” (Dunning et al., 2007) followed by a quality control and quantile normalization. LIMMA v3.2.1 was used for the further analysis of the normalized intensity values to determine the differentially expressed genes (Smyth, 2004).

All the (raw) data files have been completely uploaded to the gene expression omnibus and have been stored under accession number GSE70169.

#### Real time rtPCR

To confirm the results obtained from the microarray data in HEK293T cells, gene expression was studied in human HEK293T cell lines using Power SYBR Green RNA-to CT 1 Step Kit (Invitrogen, San Diego, CA, USA). Each reaction mixture contained 200 nM final primer concentration (primer pairs are shown in Supplemental Data Table 1) and 30 ng RNA template. All reactions were performed in triplicate on a LightCycler 480 system (Roche, Basel, Switzerland) and resulting data were analyzed by Qbase plus (Biogazelle, Ghent, Belgium). Three housekeeping genes were used each time as a reference, namely *GAPDH, RPL13A*, and *YWHAZ*.

#### Western blot analysis

For western blotting, cells were lysed using RIPA buffer (25 mM Tris-HCl (pH 7.6), 150 mM NaCl, 1% NP-40, 1% sodium deoxycholate, 0.1% SDS) (Pierce, Rockford, IL, USA) containing a PhosSTOP Phosphatase Inhibitor Cocktail Tablet (Roche), an EDTA-free protease tablet and 10 μl (25 units/μl) benzonase (70746-4 Novagen®, Merck Millipore, Darmstadt, Germany). Transfected HEK293T cells were lysed for 20′ at 4°C and centrifugated at 2000 g at 4°C to obtain protein lysates.

Proteins were electrophoretically separated and blotted onto a nitrocellulose membrane (Whatman, Kent, UK). This membrane was blocked for 1 h in 5% non-fat dry milk and afterwards incubated overnight (4°C) in one of the following primary antibodies: anti-phospho-SAPK/JNK (Thr183/Tyr185, #4668), anti-phospho-p44/42 (ERK1/2, #4370), anti-SAPK/JNK (#9252), anti-p44/42 (ERK1/2, #9102) (Cell Signaling Technologies, MA, USA), or anti-β-Actin (A5316, Sigma Aldrich, MO, USA). After washing, the membranes were incubated with either a secondary goat anti-rabbit (ab6721, Abcam, Cambridge, UK) or sheep anti-mouse (NA931, GE Healthcare, Buckingmersham, UK) antibody. Finally, the corresponding proteins were visualized using Enhanced ChemiLuminescence Western Blotting Substrate (Thermo Scientific, IL, USA).

#### MAPK inhibition

The SP600125 JNK inhibitor was used to inhibit the MAPK pathway. Twelve hours post-transfection, HEK293T cells were incubated with 25 μM for 12 h. Next, cells were collected and viability was measured by flow cytometry (CyflowML, Partec, Germany) using propidium iodide as a cell death marker.

### Gene ontology analysis

In addition to the determination of the differentially expressed genes, GO analysis was performed in order to identify enriched GO annotations. We used an open-source application, the Ontologizer, as a tool to statistically analyse the high-throughput data (Ashburner et al., 2000). A standard method for statistics, the “Term-for-Term analysis,” was used followed by Benjamini-Hochberg correction for multiple testing (Hochberg and Benjamini, 1990; Tavazoie et al., 1999). Differentially expressed genes with a corrected *p*-value below 0.05 and a log2 (fold change) of 1.5 and 0.5 in yeast and human cell lines respectively, were selected for this analysis.

## Results

To study the biological pathways of cell death induced by *DFNA5* in *S. cerevisiae*, we performed a transcriptomic study. RNA samples were collected in duplo in the mid-exponential phase and at the post-diauxic shift of yeast cells transformed with either wt*DFNA5* of mut*DFNA5*. Figure 1 shows all the comparisons studied between the different RNA samples (comparisons are labeled 1a, 1b, 2a, and 2b). Analysis of the microarray data was performed using the LIMMA package provided in R and generated four lists of differentially expressed genes. For the GO-enriched term analysis, the cut-off for adjusted *p*-values of differentially expressed genes was set to 0.05 and the cut-off of the log2 (fold change) was respectively set to 1.5 and 0.5 for yeast and human cell lines.

Investigation of the differentially expressed genes in mid-exponential phase (comparison 1a) revealed no significantly up- or down-regulated genes. Therefore, this comparison was excluded and only comparisons 1b, 2a, and 2b (shown in Figure 1) will be taken into account and described.

### Mitochondria-related processes are up-regulated in mutDFNA5 in post-diauxic shift

Comparison of the differentially expressed genes (adjusted *p* < 0.05) in the post-diauxic shift (1b) resulted in 451 significantly up-regulated genes when expressing mut*DFNA5* and using wt*DFNA5* as a reference. The top 34 differentially up-regulated genes at the post-diauxic shift are shown in Table 1. Differentially expressed genes with a log2 (fold change) equal or higher than 1.5 were selected for GO-enriched annotations, generating 85 significantly up-regulated genes, which resulted in 65 significantly up-regulated enriched GO terms.

**Table 1:** **Top 34 of the differentially up-regulated genes upon transfection of mut*****DFNA5*** **in yeast at the post-diauxic shift**.

**Gene name**	**LogFC**	**AveExpr**	**adj.p.val**	**Gene name**	**LogFC**	**AveExpr**	**adj.p.val**
MAL12	4.16	9.08	0.04	YOL047C	2.39	10.40	0.01
**COX3**	4.13	7.46	<0.01	Q0297	2.38	7.24	0.01
MAL32	3.91	8.19	0.04	MND1	2.27	7.03	0.01
ANB1	3.60	9.59	0.01	VAR1	2.23	4.18	0.02
SLZ1	3.08	6.38	<0.01	YPL141C	2.22	9.49	0.01
PRM7	3.07	11.62	0.01	HXT4	2.22	13.42	0.05
YPR077C	3.05	9.44	<0.01	YNR034W-A	2.20	13.88	0.02
PRM7	3.04	11.01	0.01	YDR034W-B	2.17	9.17	0.02
HXT7	3.02	14.80	0.03	**COX2**	2.16	6.19	0.01
YDR374C	2.94	6.69	0.02	OLI1	2.15	15.96	<0.01
YPL014W	2.89	8.59	0.02	YBL065W	2.15	6.87	0.01
**AI5_ALPHA**	2.89	10.49	0.04	SPR28	2.13	7.20	<0.01
RTA1	2.83	7.90	<0.01	**COX1**	2.12	7.77	0.01
YPR078C	2.65	7.06	<0.01	DRE2	2.05	12.58	0.01
YLR194C	2.45	13.79	0.01	YKR075C	2.01	13.64	<0.01
SUC2	2.44	11.79	0.04	YJL218W	2.00	6.92	0.04
SPO20	2.43	9.06	0.01	YLR338W	1.95	7.52	0.01

*LogFC, logarithm of fold change, up-regulation in mutDFNA5 yeast samples using wtDFNA5 as a reference; adj.p.value, p-value adjusted for multiple hypothesis testing. Genes involved in processes related to the COX activity are indicated in bold*.

Analysis of the biological, cellular, and molecular GO annotations confirmed the role of the mitochondria in mut*DFNA5*-induced cell death (Supplemental Data Table 2, indicated in bold). Analysis of the GO annotations revealed that the molecular cytochrome-c oxidase activity (GO: 0004129) related process was the most significantly up-regulated mitochondrial process. Further down the list, several biological, molecular and cellular GO processes related to mitochondrial mechanisms, such as mitochondrial ATP synthesis-coupled electron transport (GO:0042775), aerobic respiration (GO:0009060), the mitochondrial respiratory chain (GO:0005746), mitochondrial respiratory chain complex IV (GO:0005751), and oxidative phosphorylation (GO:0006119) were significantly up-regulated suggesting mitochondrial dysfunction (Supplemental Data Table 2).

Next we compared the identified GO terms with the list containing the highest differentially up-regulated genes generated by the R package LIMMA to evaluate the resemblances (Table 1). As shown in Table 1, several mitochondrial genes related to these GO processes were indeed present in the list, including *COX/1/2/3* and *AI5_ALPHA* (Table 1, bold). COX1/2/3 are three main subunits of cytochrome c oxidase, the terminal enzyme of the mitochondrial electron transport chain, encoded by the mitochondrial genome. The electron transport chain is part of mitochondrial oxidative phosphorylation providing most of the cellular ATP (Srinivasan and Avadhani, 2012). *AI5_ALPHA* is an endonuclease encoding a mobile intron of the *COXI* gene (Moran et al., 1992; Seraphin et al., 1992). Up-regulation of the main *COX* genes suggests enhanced COX activity, which has been associated with increased oxidative stress (Singh et al., 2009; Srinivasan and Avadhani, 2012). Previous data indeed demonstrated a change in redox homeostasis due to mut*DFNA5* expression in yeast (Van Rossom et al., 2012). The same study also showed increased oxidative stress measured by a dihydroethidium bromide staining (DHE). Moreover, preliminary experiments in human cell lines confirmed this and also revealed enhanced oxidative stress measured by a DHE staining (unpublished results).

In addition, two other groups of significantly enriched GO annotations could be distinguished, namely GO annotations related to catabolic and metabolic energy processes, such as oligosaccharide catabolic process (GO:0009313) or maltose catabolic processes (GO:0000025), and mechanisms related to transporter activity, such as cation (GO:0008324) and several sugar (GO:0005353 for example) transmembrane activities (Supplemental Data Table 2, respectively underlined and indicated in blue). Consistent with the previous results, comparison of these processes with the highest differentially up-regulated genes in Table 1 confirmed these identified GO terms (Table 1). Different maltose and sucrose genes like *MAL12, MAL32*, and *SUC2*, and transmembrane transporter genes like *HXT4/7* were present in the list (Table 1).

These results revealed an important role for mitochondria-related processes in mut*DFNA5* transformed yeast cells in the post-diauxic shift.

### Processes associated with glycolysis are down-regulated in *mutDFNA5* at post-diauxic shift

To investigate the significantly down-regulated processes and genes in the post-diauxic shift between wtDFNA5 and mutDFNA5, we used the same method as described in Section Mitochondria-related Processes are Up-regulated in mutDFNA5 in Post-diauxic Shift. This revealed 585 significantly down-regulated genes in cells expressing mut*DFNA5* as compared to those expressing wt*DFNA5* (adjusted *p* < 0.05). The top 34 highest differentially down-regulated genes are shown in Table 2.

**Table 2:** **Top 34 of the differentially down-regulated genes upon transfection of mut*****DFNA5*** **in yeast at the post-diauxic shift**.

**Gene name**	**LogFC**	**AveExpr**	**adj.p.val**	**Gene name**	**LogFC**	**AveExpr**	**adj.p.val**
ENO2	−3.00	16.12	0.01	ERG25	−1.77	14.05	0.05
SSA2	−2.68	14.66	0.01	YNR021W	−1.74	11.30	<0.01
VHT1	−2.54	13.48	0.02	YBT1	−1.73	12.67	0.05
DET1	−2.44	16.33	0.01	KAP122	−1.71	9.48	<0.01
RPS22A	−2.44	14.70	<0.01	**CDC19**	−1.71	16.94	0.02
**PGK1**	−2.39	16.90	0.01	YDR509W	−1.70	6.06	<0.01
**TDH3**	−2.30	15.32	0.01	RPS9B	−1.67	15.08	<0.01
**TPI1**	−2.06	17.08	0.01	GNP1	−1.64	12.19	0.01
PDC1	−2.05	16.95	0.03	FTR1	−1.61	13.04	0.04
**TDH2**	−2.02	14.26	0.01	GPD1	−1.61	13.24	0.05
YHR140W	−1.95	8.34	0.03	RPL16B	−1.61	15.38	0.01
TOS4	−1.94	10.23	0.05	YGR266W	−1.61	9.17	0.03
YRO2	−1.91	14.89	0.03	RPA43	−1.60	10.54	<0.01
FBA1	−1.91	17.14	0.01	ARC1	−1.59	13.85	0.01
RPL9A	−1.90	14.27	0.03	PGI1	−1.58	16.09	0.03
RPL22A	−1.86	14.33	0.01	RPL18B	−1.57	9.61	0.01
URA7	−1.83	12.90	<0.01	SPE3	−1.56	12.70	0.01

*LogFC, logarithm of fold change, down-regulation in mutDFNA5 yeast samples using wtDFNA5 as a reference; adj.p.value, p-value adjusted for multiple hypothesis testing. Genes involved in processes related to the glycolysis or the pentose phosphate pathways are indicated in bold*.

The significantly down-regulated biological GO annotations can be divided in two main groups (Supplemental Data Table 3). One group is related to ribosomal processes and hence translation such as cytosolic ribosome (GO:0022626) and the positive regulation of translation fidelity (GO:0045903) (Supplemental Data Table 3, indicated in bold). This down-regulation is probably due to the fact that yeast is entering the post-diauxic shift and that mutDFNA5 has a growth defect compared to wtDFNA5. Hence, this is probably not due to mut*DFNA5* expression. The second group was correlated with the biosynthesis and metabolism of glucose (GO:0006007), monosaccharide (GO:0046365) and glycolysis (GO:0006096).

Again, we compared the identified GO terms using the list containing the highest differentially down-regulated genes generated by the R package LIMMA to evaluate the resemblances (Table 2). As expected, this list contained several components associated with the glycolysis and several protein components of the small and large ribosomal subunit.

Interestingly, the list also contained several genes such as *TPI, TDH2/3, PGK1*, and *CDC19*, which are all enzymes playing a role in either the glycolytic or the pentose phosphate pathway (PPP) (Table 2 indicated in bold). *CDC19* is the yeast homolog of the human pyruvate kinase (PK) gene. Down-regulation of PK has been correlated with the activation of the PPP and the redirection of the metabolic flux from glycolysis to PPP both in human cell lines and in yeast (Ralser et al., 2007; Christofk et al., 2008; Anastasiou et al., 2011). This will enhance the anti-oxidant response and hence increase the tolerance for oxidative stress (Ralser et al., 2007; Gruning et al., 2011; Kruger et al., 2011). The down-regulation of genes involved in glycolysis and the PPP could suggest a link with oxidative stress providing a protection mechanism for mut*DFNA5*-transformed yeast cells.

### Induction of ER-related processes upon mutDFNA5 expression in yeast

In addition to the comparison of mut*DFNA5* and wt*DFNA5* in the post-diauxic shift, the modifications between mid-exponential phase and at the post-diauxic shift were investigated separately both in mut*DFNA5*-(comparison 2a Figure 1) and wt*DFNA5*-(comparison 2b Figure 1) transformed yeast cells. As both wtDFNA5 and mutDFNA5 cells demonstrated differentially expressed genes in exponential phase compared to post-diauxic shift, we expected the presence of many significantly up- or down regulated genes related to the post-diauxic shift but not solely due to mut*DFNA5* expression. Therefore, genes which were differentially expressed at the post-diauxic shift upon mut*DFNA5* transformation, but do not show any differences upon wt*DFNA5* expression in post-diauxic phase, are potentially related to mut*DFNA5*-associated processes. These were assigned as mut*DFNA5*-related changes not associated with the post-diauxic shift in yeast.

Genes with an adjusted *p*-value below 0.05 and a log2 (fold change) above 1.5 were selected for GO analysis. The GO-enriched processes significantly associated with up-regulated genes were very similar between comparison 2a and 2b and were associated with translation. GO-enriched terms significantly associated with down-regulated genes were related to ribosomes and RNA and were present both in wt*DFNA5*- and in mut*DFNA5*-transformed yeast cells. These processes were probably due to the shift to respiration and not in particular related to mut*DFNA5* (data not shown). However, three main classes could be distinguished at the post-diauxic shift. Two of them were more prominent in mut*DFNA5*-transformed yeast cells. One class was related to the biosynthesis and the metabolism of lipids (GO:0008610), such as (ergo)sterols (GO:0016126), (phyto)steroids (GO:0006694), and fatty acids (GO:0006633) (Supplemental Data Table 4, indicated in bold). The other group was associated with the ER (GO:0005783), such as the ER membrane (GO:0005789) and protein targeting to ER (GO: 0045047) (Supplemental Data Table 4, processes are underlined).

The third group which could be distinguished was related to the cytoskeleton (GO:0005856) and was more pronounced in wt*DFNA5*-transformed yeast cells. Cellularly enriched GO terms such as the microtubule cytoskeleton (GO:0015630) and the microtubule organizing center (GO:0005815) were present in this list (Supplemental Data Table 5, indicated in bold).

### Association of DFNA5 with the MAPK-related mechanisms in HEK293T cells

To further elucidate the *DFNA5*-related pathways, a microarray experiment was performed in human HEK293T cells. As described previously, mut*DFNA5* induced a growth defect in transfected HEK293T cells compared to wt*DFNA5* and control (cells transfected with an empty vector) (Op de Beeck et al., 2011). These cell death events were evident from 9 h post-transfection and peaked at 12 h (data not shown). Therefore RNA samples of HEK293T cells were collected 12 h post-transfection. A transcriptomic analysis was performed on HEK293T cells transfected with either wt*DFNA5* or mut*DFNA5*. Six biological replicates of every RNA sample were collected although one wt*DFNA5*-transfected sample did not survive quality control. Subsequent analyses, using wt*DFNA5* as a reference, were therefore performed on five wt*DFNA5*- vs. six mut*DFNA5*-transfected samples. Analysis using “Beadarray” and “LIMMA” packages available in R identified 228 significantly up- and 222 significantly down-regulated genes after correction for multiple hypothesis testing (*p* < 0.05). In addition to individual gene expression, GO analysis was performed to determine the biologically, cellularly, and molecularly enriched GO annotations linked to the differentially expressed genes.

Table 3 shows the top 34 of the significantly up-regulated genes. It contains several genes related to the MAPK pathway such as *EGR1/*2, *FOSB*, and*JUNB* (indicated in bold). Interestingly, this list also contained the *PMAIP1* gene. *PMAIP1* encodes a BH3-only protein belonging to the BCL2 protein family, a family of important regulators of apoptotic cell death related to the mitochondria. The top 34 highest down-regulated genes are shown in Table 4 and contains several genes related to protein folding such as *HSPA6, ATF3*, and *CTH* (indicated in bold, Table 4).

**Table 3:** **Top 34 of the significantly up-regulated genes in mut*****DFNA5*** **transfected HEK293T cells**.

**Gene symbol**	**logFC**	**AveExpr**	**adj.p.val**	**Gene symbol**	**logFC**	**AveExpr**	**adj.p.val**
ZCCHC12	2.63	12.07	<0.01	**PMAIP1 (2750367)**	1.00	10.76	<0.01
ETV5	1.88	9.11	<0.01	ETV4	0.99	7.71	<0.01
LOC387763	1.86	10.00	<0.01	SPRY2	0.97	9.96	<0.01
**EGR1**	1.83	11.82	<0.01	**PMAIP1 (6020598)**	0.96	9.30	<0.01
ARC	1.61	8.76	<0.01	CCNA1	0.94	7.60	<0.01
**FOSB**	1.58	8.85	<0.01	TRIM9	0.92	10.00	<0.01
KCTD12	1.40	10.32	<0.01	MAFF	0.90	7.95	<0.01
**JUNB**	1.40	9.09	<0.01	FAM84B	0.90	12.04	<0.01
ADAMTS1	1.24	9.88	<0.01	EPHA2	0.89	7.98	<0.01
**EGR2**	1.19	8.12	<0.01	CYP1B1	0.88	8.52	<0.01
**FOS**	1.18	10.17	<0.01	NDRG1	0.87	9.54	<0.01
CITED1	1.16	11.66	<0.01	SERTAD1	0.85	10.14	<0.01
MAFB	1.12	9.32	<0.01	MCL1	0.81	10.20	<0.01
GPR3	1.07	8.29	<0.01	SPRY4	0.81	7.66	<0.01
TNFRSF12A	1.06	9.77	<0.01	VGF	0.79	8.30	<0.01
TRIB1	1.04	11.60	<0.01	NEFM	0.79	11.58	<0.01
ANXA1	1.03	8.98	<0.01	GEM	0.79	7.88	<0.01

*LogFC, logarithm of fold change, down-regulation in mutDFNA5 yeast samples using wtDFNA5 as a reference; adj.p.value, p-value adjusted for multiple hypothesis testing. The array address of the specific splice variant on the microarray is provided between parentheses. Genes involved in processes related to either the MAPK pathway, cAMP response or the mitochondria are indicated in bold*.

**Table 4:** **Top 34 of the highest significantly down-regulated genes in mut*****DFNA5*** **transfected HEK293T cells**.

**Gene symbol**	**logFC**	**AveExpr**	**adj.p.val**	**Gene symbol**	**logFC**	**AveExpr**	**adj.p.val**
**HSPA6 (160092)**	−1.54	10.49	<0.01	HRK	−0.64	8.82	<0.01
DDIT4	−1.26	10.18	<0.01	C5orf13	−0.64	9.27	<0.01
CLEC2D	−0.97	9.47	<0.01	LRAP	−0.63	13.23	<0.01
ZNF256	−0.91	9.10	<0.01	CLEC2D	−0.61	14.72	<0.01
TDP1	−0.87	10.90	<0.01	GUSBL1	−0.60	10.04	<0.01
LRP5L	−0.84	8.76	<0.01	DTL	−0.60	10.17	<0.01
**CTH (60138)**	−0.80	10.40	<0.01	ZSCAN16	−0.58	8.66	<0.01
SNORD15B	−0.78	8.76	<0.01	**CTH (1470576)**	−0.58	8.75	<0.01
C17orf48	−0.76	8.45	<0.01	LOC654194	−0.58	13.17	<0.01
LOC389816	−0.73	8.57	<0.01	SNORD68	−0.58	10.17	<0.01
**HSPA6 (1710553)**	−0.71	9.28	<0.01	ZNF205	−0.57	7.83	<0.01
ZMAT3	−0.70	12.59	<0.01	ZNF416	−0.57	8.56	<0.01
PMS2L3	−0.67	8.69	<0.01	LRRC26	−0.57	8.19	<0.01
PCDHGA9	−0.66	7.61	<0.01	Hs.537645	−0.57	7.54	<0.01
**CTH (6220504)**	−0.65	8.29	<0.01	**ATF3 (4780128)**	−0.56	11.95	<0.01
C3orf34	−0.64	12.64	<0.01	ACOT2	−0.55	9.15	<0.01
FANCE	−0.64	9.35	<0.01	PCK2	−0.55	9.22	<0.01

*LogFC, logarithm of fold change, down-regulation in mutDFNA5 yeast samples using wtDFNA5 as a reference; adj.p.value, p-value adjusted for multiple hypothesis testing. The array address of the specific splice variant on the microarray is provided between parentheses. Genes involved in processes related to protein folding are indicated in bold*.

Subsequent GO analysis of the biological annotations revealed, in addition to the more general development processes, the up-regulation of the MAPK pathway (GO:0043407) and the cAMP response (GO:0051591) (Supplemental Data Table 6, indicated in bold). The response to protein folding (GO:0006986) and to topologically incorrect protein (GO:0035966) were the only two significantly down-regulated processes and both were related to protein folding (Supplemental Data Table 7). The most important genes that are involved in these processes were *HSPA6*, a heat shock protein and several chaperones proteins, such as *DNAJB1* and *DNAJB2*.

These results demonstrate the association of mut*DFNA5*-induced cell death with the MAPK pathways. The identification of processes related to protein folding supports the results in yeast in which GO terms related to protein folding and the ER were significantly associated with mutDFNA5.

### Validation of the MAPK role in DFNA5-related cell death in HEK293T cells

**Figure 2:**
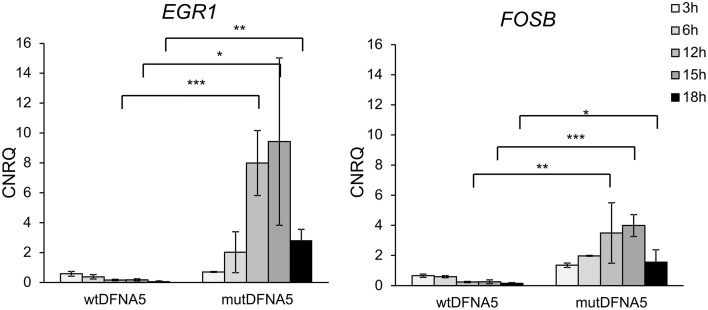
**Increased** ***EGR1*** **and** ***FOSB*** **gene expression in mut*****DFNA5*****-transfected HEK293T cells**. RNA samples were collected from HEK293T cells transfected with either wt*DFNA5* or mut*DFNA5* and gene expression was measured by real-time rtPCR. Significantly increased expression was seen in mut*DFNA5* at 12 h [p(egr1) = 0.000; p(fosB) = 0.006], 15 h [p(egr1) = 0.017; p(fosB) = 0.000] and 18 h [p(egr1) = 0.004; p(fosB) = 0.026] post-transfection. ^*^p < 0.05; ^**^p < 0.01; ^***^p < 0.001. CNRQ, calibrated normalized relative quantities.

The data generated by the transcriptomic analysis in HEK293T cells were validated by real-time rtPCR of newly collected RNA samples. *EGR1* and *FOSB* gene expression was investigated on different time-points ranging from 3 to 72 h post-transfection. As shown in Figure 2, significantly up-regulated *EGR1* and *FOSB* gene expression was observed in cells transfected with mut*DFNA5* from 12 to 18 h post transfection (*p* < 0.05) (Figure 2). Hence the data generated by the transcriptomic analyses were indeed confirmed by real time rtPCR as demonstrated by up-regulation of genes related to the MAPK pathway.

After confirmation by real time rtPCR, the significance of the activated MAPK pathway was further validated by two independent experiments. To investigate the significance of the MAPK pathway, we wondered whether inhibition of the MAPK pathway would attenuate this mutDFNA5-induced growth defect.

**Figure 3:**
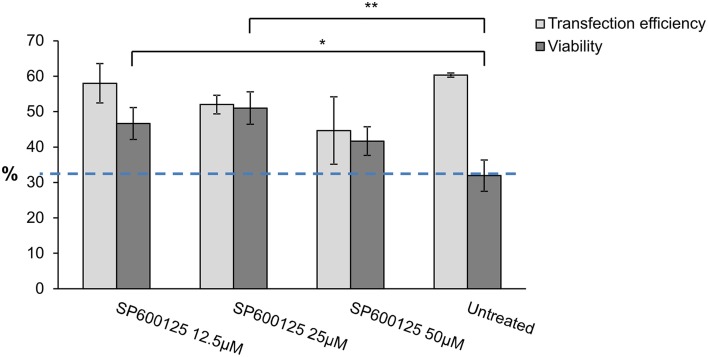
**MAPK inhibitor effect on mut*****DFNA5*** **transfected HEK293T cells**. Mut*DFNA5*-transfected HEK293T cells were pretreated with different amounts of SP600125 (JNK inhibitor). Cell viability was measured and compared to untreated mut*DFNA5*-transfected HEK293T cells. ^*^p < 0.05; ^**^p < 0.01.

Therefore, a specific JNK inhibitor, namely SP600125, was added, followed by a viability assay to determine the effect on cell survival. Different concentrations of the JNK inhibitor SP600125 were used to measure viability by flow cytometry (CyFlow ML, Partec, Germany) and these results were compared to untreated mut*DFNA5*-transfected HEK293T cells. Overnight treatment of the cells with different concentrations of SP600125 did not have any major effect on transfection efficiency, but significantly increased the viability of mut*DFNA5*-transfected cells. Although, addition of 12.5 and 25 μM SP600125 both significantly increased the viability with a *p*-value of 0.020 and 0.004 respectively SP600125 had the greatest effect with a concentration of 25 μM SP600125 since the viability was raised from 31.93 to 51.00% (Figure 3).

**Figure 4:**
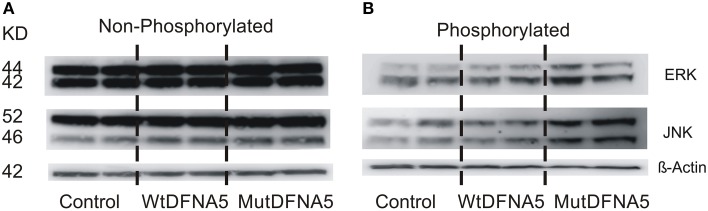
**Activation of the MAPK pathway by mutDFNA5. (A)** Western blot analysis of non- phosphorylated ERK (p42/p42) and JNK. No differences were seen in protein expression level of ERK and JNK between control, wtDFNA5 and mutDFNA5 transfected HEK293T cells. **(B)** Western blot analysis of phosphorylated, activated ERK (p42/p42) and JNK. Increased expression of JNK and to a lesser extent of ERK was seen in mutDFNA5 transfected HEK293T cells as compared to wtDFNA5 and control.

Next, to evaluate the effect of MAPK up-regulation on protein level, different MAPK proteins were studied by western blotting. There are three main MAPK pathways in human cell lines represented by the ERK, JNK, and p38 MAPK branch. Consistent with the results obtained by real time rtPCR and the viability assay, activation of the MAPK pathway proteins was also demonstrated by western blotting. Total protein lysates were collected from HEK293T cells 12 h post-transfection. Three phosphorylated (activated) and non-phosphorylated (not activated) proteins of the MAPK pathway were studied using six different antibodies. No differences were seen in the expression level of non-phosphorylated ERK and JNK (Figure 4A). Activation of JNK and to a minor extent of ERK (p42/p44) was seen upon mut*DFNA5* transfection compared to control and wt*DFNA5* (Figure 4B). The expression of p38 was also evaluated but no difference in protein expression was observed between mutDFNA5 compared to wtDFNA5 and control (data not shown). β-Actin was used as a loading control.

These results suggest that DFNA5 induces PCD mediated through activation of the MAPK pathways. Addition of a MAPK inhibitor partially attenuated the mutDFNA5-induced growth defect identifying the MAPK pathway as an early event in mutDFNA5-associated cell death.

### Comparison of the yeast microarray results with the gene expression in human cell lines

In order to study the significance of the yeast results, a comparison was made between the two microarray experiments. Human homologs of the significantly up- and down-regulated yeast genes at the post-diauxic shift (comparison 1b) were identified using Ensemble Biomart. Of the 451 significantly up- and 585 significantly down-regulated yeast genes, respectively 296 and 647 human homologs were identified. These specific human homologs were analyzed using the R package LIMMA, which generated a new list of human genes. The FC cut-off of the resulting gene list was set to FC 1.2, resulting in 16 up- and 14 down-regulated human genes (Supplemental Tables 8A,B). *TM7SF2, UCP2*, and *VPS33B*, three down-regulated human genes, were selected to verify the yeast results in human cell lines using real-time rtPCR. *UCP2* and *VPS33B* were evaluated because they were the two most down-regulated genes present in the list. UCP2, an uncoupling protein, is a mitochondrial carrier located at the mitochondrial inner membrane. Suppression of UCP2 has been linked to increased ROS production (Deng et al., 2012; Dando et al., 2013) and lifespan regulation (Andrews and Horvath, 2009; Andrews, 2010). The Vacuolar protein sorting 33 homolog (VPS33B) gene is involved in intracellular vesicle Golgi-to-lysosome transport (Pevsner et al., 1996; Lo et al., 2005). *TM7SF2* was selected based on its function in relation to the yeast results. TM7SF2 is a transmembrane protein present in the ER and associated with biosynthesis of cholesterol. In addition to its role in cholesterol synthesis, TM7SF2 appears to be involved in the inflammatory response upon cellular stress (Holmer et al., 1998; Bennati et al., 2006; Schiavoni et al., 2010; Bellezza et al., 2013).

**Figure 5:**
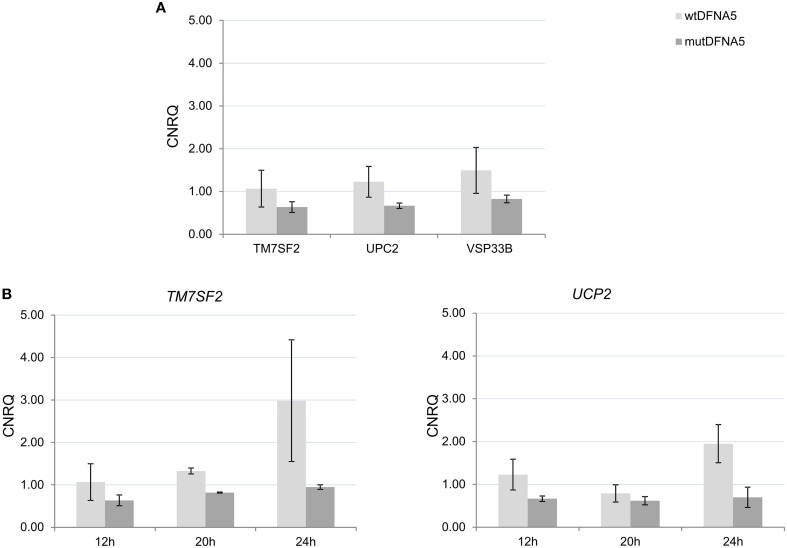
**Validation of the yeast microarray by real-time rtPCR in HEK293T cells. (A)** Gene expression 12 h post-transfection in human HEK293T cells of three selected genes. **(B)** Gene expression 12, 20, and 24 h post-transfection with either wt*DFNA5* or mutant *DFNA5*. *UCP2*, uncoupling protein 2 (mitochondrial, proton carrier); *TM7SF2*, transmembrane 7 superfamily member 2; *VPS33B*, vacuolar protein sorting 33 homolog. Light gray, wt*DFNA5*; Dark gray, mut*DFNA5*. ^*^p < 0.05. CNRQ, calibrated normalized relative quantities.

To verify the yeast results in human cell lines, RNA was collected from HEK293T cells transfected with either wt*DFNA5* or mut*DFNA5* at different time-points starting at 12 h after transfection as this was the time-point of the human microarray experiment. The *TM7SF2, UCP2*, and *VPS33B* genes had a fold change of respectively 1.23, 1.31, and 1.27 on the microarray. Real-time rtPCR on RNA samples 12 h post-transfection confirmed these microarray results as all three genes were down-regulated in mut*DFNA5* compared to wt*DFNA5* (Figure 5A) with fold changes comparable to the microarray (respectively 1.35, 1.48, and 1.59). Although not significantly, these three genes were down-regulated in HEK293T cells at 12 h after transfection (Figure 5A). The down-regulation was still present at 20 h, peaked at 24 h after transfection and was even significant for TM7SF2 (p:0.01) and UCP2 (p:0.07) at respectively 20 h and 24 h after transfection (Figure 5B). Due to this down-regulation, we can conclude that there are some similarities between the yeast and the HEK293T microarray. Differences are seen when looking at the individual genes, but upon study of the different pathways a role for processes related to protein folding were seen in both model systems.

## Discussion

This study further investigated the biological effects induced by *DFNA5* expression in *Saccharomyces cerevisiae* and in human HEK293T cells. A previous study in human cell lines revealed that mut*DFNA5* is a cell death-inducing gene (Op de Beeck et al., 2011). As described previously (Van Rossom et al., 2012), transformation of mut*DFNA5* in yeast resulted in a growth defect associated with four different mitochondria-related proteins. This study was the first to establish a possible link between the mitochondria and DFNA5. In addition, the 2012 study observed that the protein quality control system, responsible for correct protein folding and degradation, had problems coping with mutDFNA5. A failing protein quality control system could indicate the presence of ER stress, as the ER can be involved in protein folding and degradation.

This study confirmed the involvement of mitochondria-related processes upon expression of mut*DFNA5* in *Saccharomyces cerevisiae*, especially the ATP-coupled electron transport. Several genes related to either the glycolysis and the PPP were significantly down-regulated upon mut*DFNA5* transformation in yeast. Furthermore, we show that the JNK and ERK MAPK pathways are activated *in vitro* after transfection of mut*DFNA5* in HEK293T cells and that inhibition of this pathway is able to partially attenuate the resulting cell death. Additionally, this study also revealed an association of GO annotations related to the ER and protein folding in both model organisms.

### Oxidative stress and hearing loss

The up-regulation of different cytochrome c oxidase (*COX*) genes revealed a potential association of the mutDFNA5-related cell death mechanisms with oxidative stress in yeast. Increased oxidative stress was already demonstrated in yeast (Van Rossom et al., 2012) and was later confirmed in human cell lines (unpublished results). Enhanced COX activity has indeed been associated with increased oxidative stress (Singh et al., 2009; Srinivasan and Avadhani, 2012). COX is the rate limiting enzyme of respiration which regulates the bio-energetic status of the cell. Dependent on the cellular energetic requirements, the COX activity can be rapidly adapted. The ratio of ATP/ADP is one of the regulators of the COX activity. High ADP levels or ATP utilization will increase the enzyme activity and stimulate the respiration (Napiwotzki and Kadenbach, 1998; Arnold and Kadenbach, 1999). Activation of the COX activity could result in higher oxidative stress generated at the mitochondria.

Additionally, the correlation of mutDFNA5 with several genes of the PPP can also be linked to oxidative stress in yeast as the PPP plays a major role in the anti-oxidant response. Reduced expression of *CDC19*, the yeast homolog of PK, and of *TPI* has been correlated with the activation of the PPP and the redirection of the metabolic flux from glycolysis to PPP both in human cell lines and in yeast (Christofk et al., 2008; Gruning et al., 2011, 2014). This study revealed reduced gene expression of both *TPI* and *CDC19*, indicating a shift in redox sensing in eukaryotes mediating a fast response to oxidative stress. Activation of the PPP is correlated with the inhibition of ROS accumulation and enhancement of the anti-oxidant response upon shift from fermentation to respiration. PPP activation will enhance the anti-oxidant response and hence increase the tolerance for oxidative stress (Ralser et al., 2007; Gruning et al., 2011; Kruger et al., 2011). These data clearly demonstrate a change in redox homeostasis due to mut*DFNA5* expression which was shown previously in yeast (Van Rossom et al., 2012).

Furthermore, enhanced oxidative stress is often related to a failing protein quality control system (Davies, 2001; Shang et al., 2001; Bender et al., 2010, 2011; Shang and Taylor, 2011). This possibility was already suggested by the proteolytic degradation previously seen upon wt*DFNA5* transformation, but which was absent in mut*DFNA5*-transformed yeast cells (Van Rossom et al., 2012). MutDFNA5 seemed to escape this quality control system, in contrast to wtDFNA5 which was subject to the normal clearance system. Moreover, the authors suggested a possible link between protein degradation and the mitochondria in mutDFNA5-induced cell death as yeast seemed to have problems with proper mutDFNA5 protein turnover. The same link was confirmed in both model organisms used in the current study.

### Role of ER stress in the pathology of hearing loss

The increase in mitochondrial metabolism seen in this study and the decrease of protein folding processes, can also be explained by the presence of cellular ER stress leading to an unfolded protein response. Mitochondria and ER form an interconnected network which is important for several biological processes mediating an adaptive response under various cellular stress conditions (De Brito and Scorrano, 2010; Marchi et al., 2014). The association between mutDFNA5 and the GO terms related to lipid metabolism, protein targeting to ER and the ER membrane, suggest the presence of ER stress (Schroder, 2008). Mitochondria depend on the ER for the import of several proteins and lipids and for Ca^2+^ exchange involved in cell death and mitochondrial metabolism (Sauner and Levy, 1971; Zecchini et al., 2007; Stiban et al., 2008; Wiel et al., 2014). Enhanced Ca^2+^ supply will increase ATP production and mitochondrial respiration, processes which were indeed both up-regulated at the post-diauxic shift upon mut*DFNA5* expression. Prolonged enhancement however will eventually have a detrimental effect on the mitochondria.

Despite limited knowledge correlating mutDFNA5 with ER stress, a correlation has been established between ER stress and certain causes of HL. Ototoxicity (HL due to the use of pharmaceuticals such as aminoglycoside antibiotics and platinum-based chemotherapeutics) was shown to be correlated with ER stress-dependent pathways. Certain pain relievers, contributing to tinnitus and progressive bilateral sensorineural HL, were shown to induce ROS overproduction, altered ER morphology and changes in ER stress markers, such as CHOP (Kalinec et al., 2014).

Taken together, the previous observation of the importance of the mitochondria in mutDFNA5-related cell death and the known correlation between the ER and the mitochondria points to a potential role for the ER in DFNA5-induced cell death. The failing of the protein quality control system in mutDFNA5 suggests the involvement of the ER, but this remains unclear at this moment and needs to be further investigated in the future.

### Contribution of the mitochondria in MAPK-related cell death

In addition to the mitochondria, the MAPK pathways seem to play a prominent role in mutDFNA5-induced cell death in HEK293T cells. The link between the mitochondria and MAPK in DFNA5-related cell death is unknown at this moment, but several mitochondria-MAPK correlations have been described. It is known that MAPK pathways can be induced by ROS production generated by the mitochondria (Chambers and Lograsso, 2011). As increased oxidative stress has been shown in an earlier study in yeast and in human cell lines (unpublished results), this can provide a direct link between these two processes. This could suggest that ROS activates the MAPK pathway and hence lays up-stream of the MAPK pathway in DFNA5-induced cell death. However, we showed in this study that a specific MAPK inhibitor was able to attenuate the cell death and we have unpublished results showing that several anti-oxidants did not inhibit DFNA5-induced cell death. This implies that ROS is either a secondary event not directly causing cell death, or that the activation of MAPK is an early event in the cell death process, up-stream of ROS production. Furthermore, comparison of yeast and the human microarray results identified *UCP2*, a gene associated with the mitochondria, which showed reduced gene expression in *mutDFNA5* compared to *wtDFNA5*. Interestingly, mitochondrial stress has been linked to the down-regulation of *UCP2* by activation of the MAPK pathway and of JNK activation in particular (Emre et al., 2007; Selimovic et al., 2008). *UCP2* reduction was an early event required for the amplification of the activated MAPK pathway enabling mitochondrial ROS production (Emre et al., 2007; Basu Ball et al, 2011). This down-regulation enables mitochondrial ROS production providing the amplification loop stimulating the MAPK pathway. UCP2 can therefore provide the link between MAPK and the mitochondria, regulating the ROS production, a feature deregulated in both model organisms.

However, we did not observe an up-regulation of MAPK-related pathways in yeast. This could be explained by the differences in timing between the experiments. For human cell lines, the experiment was performed 12 h post-transfection, while yeast RNA was collected at the post-diauxic shift.

In conclusion, this study confirms the role of the mitochondria in mutDFNA5-induced toxicity in yeast. Additionally, it shows that mutDFNA5-induced cell death is mediated by the MAPK pathway, especially through the ERK and the JNK branch. Inhibition of this pathway could significantly enhance the cell viability of mut*DFNA5*-transfected HEK293T cells which suggests the importance of this signaling cascade for DFNA5. How the MAPK pathways perform their role in cell death and the connection with DFNA5-related mechanisms remains uncertain at this moment but is a good starting for future studies. Future studies further unraveling the DFNA5-induced cell death mechanism are important as they may lead to new insights in the involvement of mitochondria in HL and have the potential to lead to new future therapies.

### Conflict of interest statement

The funders had no role in study design, data collection and analysis, decision to publish, or preparation of the manuscript. Joris Winderickx declares that he is co-founder of the KU Leuven spin-off companies reMYND and ADxNeuroSciences, but this did not influence study design, data collection, analysis, publication or the preparation of the manuscript. The authors declare that the research was conducted in the absence of any commercial or financial relationships that could be construed as a potential conflict of interest.
