# Supplementary material for: The deafness gene DFNA5 induces programmed cell death through mitochondria and MAPK-related pathways
Source: Front Cell Neurosci. 2015 Jul 16;9:231. doi: 10.3389/fncel.2015.00231 (PMC4504148; doi:10.3389/fncel.2015.00231)
Supplement: Supplementary file 1 [file Table1.PDF]

Table 1: Primer sequences for RT-PCR. HHG: housekeeping genes used for normalization. Fw, forward; Rv, reverse sequence.

Gene	Fw_sequence	Rv_sequence
TM7SF2	TCT ACA TGA AGG CGC AGG TAG C	AGT CGT AAA TCG GAT TGC CTG AG
UCP2	TCT CCC AAT GTT GCT CGT AAT GCC	AAG TGG CAA GGG AGG TCA TCT GTC
VPS33B	TGT GGA TCT GCT GAG CAT GGA AC	TCC AAC GCT GAT CTC CTT CCA G
FOSB	GAG AGC TGG TAG TTA GTA GCA TGT GA	AAT TCC AAT AAT GAA CCC AAT AGA TTA
EGR1	GCA CCT GAC CGC AGA GTC TT	AGT GGT TTG GCT GGG GTA ACT
GAPD_HHG	TGC ACC ACC AAC TGC TTA GC	GGC ATG GAC TGT GGT CAT GAG
RPL13A_HHG	CCT GGA GTT TTC TTT CCA GAG	TTG AGA ACC TCT GTG TAT TTG
YWHAZ_HHG	ACT TTT GGT ACA TTG TGG CTT	CCG CCA GGA CAA ACC AGT AT
