# Supplementary material for: The deafness gene DFNA5 induces programmed cell death through mitochondria and MAPK-related pathways
Source: Front Cell Neurosci. 2015 Jul 16;9:231. doi: 10.3389/fncel.2015.00231 (PMC4504148; doi:10.3389/fncel.2015.00231)
Supplement: Supplementary file 2 [file Table2.PDF]

**Table 2: condensed list of all the significantly up-regulated GO annotations at the post-diauxic shift.** Population term: the number of genes in the whole yeast population set (5640 genes) that are annotated to the GO term in question. Study term: the number of genes in the study set that is annotated to the GO term in question. The study set contained 75 significantly up-regulated genes with a log2(FC) < 1.5. Wt*DFNA5* transformed yeast cells were used as a reference. GO enriched terms related to mitochondria are indicated in bold, to transporter activity is indicated in blue and GO terms related to energy metabolism are underlined. '… 'denotes a gap in the list due to space restriction. adj.p.value: p-value adjusted for multiple hypothesis testing.

| ID | Pop.term | Study.term | Adj.p.val | Name |
|---|---|---|---|---|
| GO:0016676 | 19 | 6 | <0.01 | oxidoreductase activity, acting on a heme group of donors, oxygen as acceptor |
| GO:0016675 | 19 | 6 | <0.01 | oxidoreductase activity, acting on a heme group of donors |
| GO:0004129 | 19 | 6 | <0.01 | **cytochrome-c oxidase activity** |
| GO:0015002 | 19 | 6 | <0.01 | heme-copper terminal oxidase activity |
| GO:0015078 | 68 | 9 | <0.01 | hydrogen ion transmembrane transporter activity |
| GO:0015077 | 86 | 9 | <0.01 | monovalent inorganic cation transmembrane transporter activity |
| … | … | … | … | … |
| GO:0009055 | 33 | 5 | 0.01 | **electron carrier activity** |
| GO:0009313 | 18 | 4 | 0.01 | oligosaccharide catabolic process |
| GO:0045333 | 108 | 8 | 0.01 | **cellular respiration** |
| GO:0009060 | 85 | 7 | 0.01 | **aerobic respiration** |
| GO:0005985 | 8 | 3 | 0.01 | sucrose metabolic process |
| GO:0005987 | 8 | 3 | 0.01 | sucrose catabolic process |
| GO:0000025 | 2 | 2 | 0.01 | maltose catabolic process |
| … | … | … | … | … |
| GO:0008645 | 24 | 4 | 0.01 | hexose transport |
| GO:0046906 | 24 | 4 | 0.01 | tetrapyrrole binding |

**Table 2 continued: Condensed list of all the GO annotations significantly associated with the up-regulated genes at the post-diauxic shift**

| ID | Pop.term | Study.term | Adj.p.val | Name |
|---|---|---|---|---|
| GO:0015749 | 24 | 4 | 0.01 | monosaccharide transport |
| GO:0008324 | 170 | 9 | 0.02 | cation transmembrane transporter activity |
| GO:0005746 | 27 | 4 | 0.02 | **mitochondrial respiratory chain** |
| GO:0015926 | 27 | 4 | 0.02 | **glucosidase activity** |
| GO:0006123 | 12 | 3 | 0.02 | **mitochondrial electron transport, cytochrome c to oxygen** |
| GO:0005751 | 12 | 3 | 0.02 | **mitochondrial respiratory chain complex IV** |
| GO:0045277 | 12 | 3 | 0.02 | **respiratory chain complex IV** |
| GO:0042773 | 28 | 4 | 0.02 | **ATP synthesis coupled electron transport** |
| GO:0042775 | 28 | 4 | 0.02 | **mitochondrial ATP synthesis coupled electron transport** |
| GO:0006119 | 29 | 4 | 0.02 | **oxidative phosphorylation** |
| GO:0000023 | 13 | 3 | 0.02 | maltose metabolic process |
| GO:0005215 | 443 | 15 | 0.02 | transporter activity |
| GO:0022857 | 349 | 13 | 0.02 | transmembrane transporter activity |
| GO:0005984 | 30 | 4 | 0.02 | disaccharide metabolic process |
| GO:0022904 | 30 | 4 | 0.02 | **respiratory electron transport chain** |
| GO:0022900 | 31 | 4 | 0.02 | **electron transport chain** |
| GO:0070469 | 32 | 4 | 0.02 | **respiratory chain** |
| GO:0022891 | 314 | 12 | 0.02 | substrate-specific transmembrane transporter activity |
| GO:0005353 | 15 | 3 | 0.02 | fructose transmembrane transporter activity |
| GO:0015578 | 15 | 3 | 0.02 | mannose transmembrane transporter activity |
| … | … | … | … | … |
| GO:0015146 | 4 | 2 | 0.02 | pentose transmembrane transporter activity |
| … | … | … | … | … |

**Table 2 continued: Condensed list of all the GO annotations significantly associated with the up-regulated genes at the post-diauxic shift**

| ID | Pop.term | Study.term | Adj.p.val | Name |
|---|---|---|---|---|
| GO:0015145 | 17 | 3 | 0.03 | monosaccharide transmembrane transporter activity |
| GO:0044455 | 166 | 8 | 0.03 | **mitochondrial membrane part** |
| GO:0005506 | 38 | 4 | 0.03 | iron ion binding |
| GO:0044724 | 95 | 6 | 0.03 | single-organism carbohydrate catabolic process |
| GO:0044275 | 39 | 4 | 0.03 | cellular carbohydrate catabolic process |
| … | … | … | … | … |
| GO:0051119 | 20 | 3 | 0.04 | sugar transmembrane transporter activity |
| GO:0008643 | 43 | 4 | 0.04 | carbohydrate transport |
| GO:0016052 | 105 | 6 | 0.04 | carbohydrate catabolic process |
| GO:0009311 | 44 | 4 | 0.04 | oligosaccharide metabolic process |
