# Supplementary material for: The deafness gene DFNA5 induces programmed cell death through mitochondria and MAPK-related pathways
Source: Front Cell Neurosci. 2015 Jul 16;9:231. doi: 10.3389/fncel.2015.00231 (PMC4504148; doi:10.3389/fncel.2015.00231)
Supplement: Supplementary file 3 [file Table3.PDF]

Table 3: List of all the significantly down-regulated GO annotations at the post-diauxic shift. Population term: the number of genes in the yeast population set (5640 yeast genes) that are annotated to the GO term in question. Study term: the number of genes in the study set that is annotated to the GO term in question. The study set contained 36 significantly down-regulated genes with a log2(FC) < 1.5. Wt*DFNA5* transformed yeast cells were used as a reference. GO enriched terms related to translation are indicated in bold. adj.p.value: p-value adjusted for multiple hypothesis testing.

ID	Pop.term	Study.term	Adj.p.value	Name
GO:0006007	52	10	<0.01	glucose catabolic process
GO:0019320	59	10	<0.01	hexose catabolic process
GO:0046365	65	10	<0.01	monosaccharide catabolic process
GO:0006096	32	8	<0.01	glycolysis
GO:0044724	95	10	<0.01	single-organism carbohydrate catabolic process
GO:0006006	98	10	<0.01	glucose metabolic process
GO:0016052	105	10	<0.01	carbohydrate catabolic process
GO:0006094	32	7	<0.01	gluconeogenesis
GO:0019319	33	7	<0.01	hexose biosynthetic process
GO:0046364	34	7	<0.01	monosaccharide biosynthetic process
GO:0019318	115	10	<0.01	hexose metabolic process
GO:0005996	121	10	<0.01	monosaccharide metabolic process
GO:0006091	189	9	<0.01	generation of precursor metabolites and energy
GO:0016051	97	7	<0.01	carbohydrate biosynthetic process
GO:0005886	454	13	<0.01	plasma membrane
GO:0005975	343	11	<0.01	carbohydrate metabolic process
GO:0044723	298	10	<0.01	single-organism carbohydrate metabolic process
GO:0044445	235	9	<0.01	cytosolic part
GO:0016861	8	3	<0.01	intramolecular oxidoreductase activity, interconverting aldoses and ketoses
GO:0005829	531	12	<0.01	cytosol
GO:0022626	168	7	<0.01	**cytosolic ribosome**
GO:0006098	15	3	<0.01	pentose-phosphate shunt
GO:0071944	650	13	<0.01	cell periphery

Table 3 continued: List of all the significantly down-regulated GO annotations at the post-diauxic shift.

ID	Pop.term	Study.term	Adj.p.value	Name
GO:0004365	3	2	<0.01	glyceraldehyde-3-phosphate dehydrogenase (NAD+) (phosphorylating) activity
GO:0044712	491	11	<0.01	single-organism catabolic process
GO:0006740	19	3	0.01	NADPH regeneration
GO:0016860	20	3	0.01	intramolecular oxidoreductase activity
GO:0046496	50	4	0.01	nicotinamide nucleotide metabolic process
GO:0019362	51	4	0.01	pyridine nucleotide metabolic process
GO:0003735	214	7	0.01	**structural constituent of ribosome**
GO:0006739	22	3	0.01	NADP metabolic process
GO:0051287	25	3	0.01	NAD binding
GO:0044391	229	7	0.01	**ribosomal subunit**
GO:0045903	6	2	0.01	**positive regulation of translational fidelity**
GO:0072524	62	4	0.01	pyridine-containing compound metabolic process
GO:0006733	65	4	0.01	oxidoreduction coenzyme metabolic process
GO:1901576	1945	22	0.02	organic substance biosynthetic process
GO:0044711	817	13	0.02	single-organism biosynthetic process
GO:0009058	1973	22	0.02	biosynthetic process
GO:0006732	144	5	0.04	coenzyme metabolic process
GO:1901575	777	12	004.	organic substance catabolic process
GO:0022625	89	4	0.04	**cytosolic large ribosomal subunit**
GO:0006090	13	2	0.05	pyruvate metabolic process
