# Supplementary material for: The deafness gene DFNA5 induces programmed cell death through mitochondria and MAPK-related pathways
Source: Front Cell Neurosci. 2015 Jul 16;9:231. doi: 10.3389/fncel.2015.00231 (PMC4504148; doi:10.3389/fncel.2015.00231)
Supplement: Supplementary file 4 [file Table4.PDF]

Table 4: Condensed list of significantly down-regulated GO terms of mut*DFNA5* transformed yeast cells in stationary phase compared to mid-exponential phase. Population term: the number of genes in the yeast population set (5640 yeast genes) that are annotated to the GO term in question. Study term: The numbers of genes in the study set that are annotated to the GO term in question. The study set contained 541 significantly up-regulated genes with a log2(FC) < 1.5. The first 50 GO terms were left out, as they were all related to ribosomal processes and similar to wt*DFNA5* down-regulated processes. GO terms related to lipid metabolism are indicated in bold, GO terms associated to the endoplasmic reticulum/protein transport are underlined. '… 'denotes a gap in the list due to space restriction. adj.p.value: p-value adjusted for multiple hypothesis testing.

ID	Pop.term	Study.term	Adj.p.value	Name
…	…	…	…	…
GO:0006696	29	13	<0.01	**ergosterol biosynthetic process**
GO:0044108	29	13	<0.01	**cellular alcohol biosynthetic process**
GO:0016129	29	13	<0.01	**phytosteroid biosynthetic process**
GO:0046165	81	23	<0.01	**alcohol biosynthetic process**
GO:0006694	39	15	<0.01	**steroid biosynthetic process**
GO:0016126	39	15	<0.01	**sterol biosynthetic process**
GO:0071704	3529	388	<0.01	organic substance metabolic process
GO:1901617	89	24	<0.01	organic hydroxy compound biosynthetic process
GO:0008202	50	17	<0.01	**steroid metabolic process**
GO:0016125	50	17	<0.01	**sterol metabolic process**
GO:0008204	31	13	<0.01	**ergosterol metabolic process**
GO:0016128	31	13	<0.01	**phytosteroid metabolic process**
GO:0009277	97	25	<0.01	fungal-type cell wall
…	…	…	…	…
GO:0090502	47	15	<0.01	RNA phosphodiester bond hydrolysis, endonucleolytic
GO:0006629	308	52	<0.01	**lipid metabolic process**
GO:0005829	531	79	<0.01	cytosol
GO:0006633	33	12	<0.01	**fatty acid biosynthetic process**
GO:0009987	4554	471	<0.01	cellular process
…	…	…	…	…

Table 4 continued: Condensed list of significantly down-regulated GO terms of mut*DFNA5* transformed yeast cells in stationary phase compared to mid-exponential phase

ID	Pop.term	Study.term	Adj.p.value	Name
GO:0034641	2046	239	<0.01	cellular nitrogen compound metabolic process
GO:0005783	478	72	<0.01	endoplasmic reticulum
GO:1901070	6	5	<0.01	guanosine-containing compound biosynthetic process
GO:0046037	6	5	<0.01	GMP metabolic process
GO:0044432	379	60	<0.01	endoplasmic reticulum part
…	…	…	…	…
GO:1901137	223	40	<0.01	carbohydrate derivative biosynthetic process
GO:0042175	366	58	<0.01	**nuclear outer membrane-endoplasmic reticulum membrane network**
GO:0006753	310	51	<0.01	nucleoside phosphate metabolic process
…	…	…	…	…
GO:0005789	353	56	<0.01	endoplasmic reticulum membrane
GO:0030497	4	4	<0.01	**fatty acid elongation**
GO:0006725	1974	230	<0.01	cellular aromatic compound metabolic process
…	…	…	…	…
GO:0072599	38	12	<0.01	establishment of protein localization to endoplasmic reticulum
GO:0045047	38	12	<0.01	protein targeting to ER
GO:1901135	411	62	<0.01	carbohydrate derivative metabolic process
GO:0005844	33	11	<0.01	polysome
GO:0006913	173	32	<0.01	nucleocytoplasmic transport
GO:0022618	144	28	0.01	ribonucleoprotein complex assembly
GO:0006066	130	26	0.01	**alcohol metabolic process**
…	…	…	…	…
GO:0043001	11	6	0.01	Golgi to plasma membrane protein transport
GO:0046365	65	16	0.01	monosaccharide catabolic process
…	…	…	…	…
GO:0072659	17	7	0.01	protein localization to plasma membrane

Table 4 continued: Condensed list of significantly down-regulated GO terms of mut*DFNA5* transformed yeast cells in stationary phase compared to mid-exponential phase

ID	Pop.term	Study.term	Adj.p.value	Name
GO:0090002	17	7	0.01	establishment of protein localization to plasma membrane
…	…	…	…	…
GO:0004312	9	5	0.02	**fatty acid synthase activity**
GO:0009147	9	5	0.02	pyrimidine nucleoside triphosphate metabolic process
GO:0019319	33	10	0.02	hexose biosynthetic process
GO:0070972	45	12	0.02	protein localization to endoplasmic reticulum
