# Supplementary material for: The deafness gene DFNA5 induces programmed cell death through mitochondria and MAPK-related pathways
Source: Front Cell Neurosci. 2015 Jul 16;9:231. doi: 10.3389/fncel.2015.00231 (PMC4504148; doi:10.3389/fncel.2015.00231)
Supplement: Supplementary file 5 [file Table5.PDF]

Table 5: Condensed list of significantly down-regulated GO terms of wt*DFNA5* transformed yeast cells in stationary phase compared to mid-exponential phase. Population term: the number of genes in the yeast population set (5640 yeast genes) that are annotated to the GO term in question. Study term: The numbers of genes in the study set that are annotated to the GO term in question. The study set contained 331 significantly up-regulated genes with a log2(FC) < 1.5. First 40 GO terms were left out, as they were all related to ribosomal processes and similar to mut*DFNA5* down-regulated processes. GO terms related to cytoskeleton/microtubuli are indicated in bold. adj.p.value: p-value adjusted for multiple hypothesis testing.

ID	Pop.term	Study.term	Adj.p.value	Name
…	…	…	…	…
GO:0005856	239	34	<0.01	**Cytoskeleton**
GO:0031974	1070	98	<0.01	membrane-enclosed lumen
GO:0008610	187	29	<0.01	lipid biosynthetic process
GO:0022613	427	50	<0.01	ribonucleoprotein complex biogenesis
GO:0044430	224	32	<0.01	**cytoskeletal part**
GO:0000226	106	20	<0.01	**microtubule cytoskeleton organization**
GO:0005874	64	15	<0.01	**Microtubule**
GO:0005935	166	26	<0.01	cellular bud neck
GO:0071944	650	66	<0.01	cell periphery
GO:0048610	406	47	<0.01	cellular process involved in reproduction
GO:0000922	82	17	<0.01	spindle pole
GO:0022414	254	34	<0.01	reproductive process
GO:0005828	6	5	<0.01	**kinetochore microtubule**
GO:0005815	75	16	<0.01	**microtubule organizing center**
GO:0005816	75	16	<0.01	spindle pole body
GO:0044764	122	21	<0.01	multi-organism cellular process
GO:1901617	89	17	<0.01	organic hydroxy compound biosynthetic process
GO:0005880	7	5	<0.01	**nuclear microtubule**
GO:0032040	48	12	<0.01	small-subunit processome
GO:0046165	81	16	<0.01	alcohol biosynthetic process
GO:0005788	16	7	<0.01	endoplasmic reticulum lumen
GO:0034660	423	46	<0.01	ncRNA metabolic process
GO:0044085	1017	89	<0.01	cellular component biogenesis

Table 5 continued: Condensed list of significantly down-regulated GO terms of wt*DFNA5* transformed yeast cells in stationary phase compared to mid-exponential phase

ID	Pop.term	Study.term	Adj.p.value	Name
GO:0019953	219	29	<0.01	sexual reproduction
GO:0044703	219	29	<0.01	multi-organism reproductive process
GO:0005200	29	9	<0.01	**structural constituent of cytoskeleton**
GO:0005876	23	8	<0.01	spindle microtubule
GO:0007051	44	11	<0.01	spindle organization
GO:0007052	38	10	<0.01	mitotic spindle organization
GO:0044452	71	14	<0.01	nucleolar part
GO:0071840	2122	158	<0.01	cellular component organization or biogenesis
GO:0007010	232	29	<0.01	**cytoskeleton organization**
GO:0016538	26	8	<0.01	cyclin-dependent protein serine/threonine kinase regulator activity
GO:0044815	20	7	<0.01	DNA packaging complex
GO:0000027	34	9	<0.01	ribosomal large subunit assembly
GO:0000462	95	16	<0.01	maturation of SSU-rRNA from tricistronic rRNA transcript (SSU-rRNA, 5.8S rRNA, LSU-rRNA)
GO:0044450	15	6	<0.01	**microtubule organizing center part**
