# Supplementary material for: The deafness gene DFNA5 induces programmed cell death through mitochondria and MAPK-related pathways
Source: Front Cell Neurosci. 2015 Jul 16;9:231. doi: 10.3389/fncel.2015.00231 (PMC4504148; doi:10.3389/fncel.2015.00231)
Supplement: Supplementary file 6 [file Table6.PDF]

Table 6: Significantly up-regulated biological GO terms in HEK293T cells using wt*DFNA5* as a reference. Population term: the number of genes in the yeast population set (26640 human genes) that are annotated to the GO term in question. Study term: the number of genes in the study set that is annotated to the GO term in question. The study set contained 79 significantly up-regulated genes with a log2(FC) < 0.5. GO terms related to the cAMP response and the MAPK pathway are indicated in bold. adj.p.value: p-value adjusted for multiple hypothesis testing.

Biological GO term	Pop.term	Study.term	Adj.p.value	Name
GO:0007275	4169	45	<0.01	multicellular organismal development
GO:0032502	4721	47	<0.01	developmental process
GO:0044767	4669	46	<0.01	single-organism developmental process
GO:0032501	5653	50	<0.01	multicellular organismal process
GO:0044707	5444	49	<0.01	single-multicellular organism process
GO:0051591	86	8	<0.01	**response to cAMP**
GO:0048856	4195	41	<0.01	anatomical structure development
GO:0048513	2587	31	<0.01	organ development
GO:0048731	3621	37	<0.01	system development
GO:0050794	7865	56	<0.01	regulation of cellular process
GO:0048523	3017	33	<0.01	negative regulation of cellular process
GO:0046683	114	8	<0.01	response to organophosphorus
GO:0009888	1395	22	<0.01	tissue development
GO:0080090	4512	41	<0.01	regulation of primary metabolic process
GO:0048585	948	18	<0.01	negative regulation of response to stimulus
GO:0050789	8324	57	<0.01	regulation of biological process
GO:0048519	3313	34	<0.01	negative regulation of biological process
GO:0014074	129	8	<0.01	response to purine-containing compound
GO:0009893	2193	27	<0.01	positive regulation of metabolic process
GO:0010604	1992	25	<0.01	positive regulation of macromolecule metabolic process
GO:0060255	4267	38	<0.01	regulation of macromolecule metabolic process
GO:0043407	65	6	<0.01	**negative regulation of MAP kinase activity**
GO:1902532	287	10	<0.01	negative regulation of intracellular signal transduction

Table 6 continued: Significantly up-regulated biological GO terms in HEK293T cells using wt*DFNA5* as a reference

Biological GO term	Pop.term	Study.term	Adj.p.value	Name
GO:0010605	1464	21	<0.01	negative regulation of macromolecule metabolic process
GO:0043409	111	7	<0.01	**negative regulation of MAPK cascade**
GO:0006469	166	8	<0.01	negative regulation of protein kinase activity
GO:0031325	2083	25	<0.01	positive regulation of cellular metabolic process
GO:0065007	8823	57	<0.01	biological regulation
GO:0031323	4611	39	<0.01	regulation of cellular metabolic process
