# Supplementary material for: The deafness gene DFNA5 induces programmed cell death through mitochondria and MAPK-related pathways
Source: Front Cell Neurosci. 2015 Jul 16;9:231. doi: 10.3389/fncel.2015.00231 (PMC4504148; doi:10.3389/fncel.2015.00231)
Supplement: Supplementary file 7 [file Table7.PDF]

Table 7: Significantly down-regulated biological GO terms in HEK293T cells using wt*DFNA5* as a reference. Population term: the number of genes in the yeast population set (26640 human genes) that are annotated to the GO term in question. Study term: the number of genes in the study set that is annotated to the GO term in question. The study set contained 50 significantly up-regulated genes with a log2(FC) < 0.5. adj.p.value: p-value adjusted for multiple hypothesis testing.

**Biological GO term**	**Pop.term**	**Study.term**	**Adj.p.value**	**name**
GO:0006986	134	5	0.01	response to unfolded protein
GO:0035966	142	5	0.01	response to topologically incorrect protein
