# Supplementary material for: The deafness gene DFNA5 induces programmed cell death through mitochondria and MAPK-related pathways
Source: Front Cell Neurosci. 2015 Jul 16;9:231. doi: 10.3389/fncel.2015.00231 (PMC4504148; doi:10.3389/fncel.2015.00231)
Supplement: Supplementary file 8 [file Table8.PDF]

Table 8A: The highest down-regulated genes in mut*DFNA5* transfected HEK293T cells based on the significant yeast down-regulated genes. Cut-off of fold change was set at 1.2.

Gene symbol	FC_absolute
UCP2	1,307405838
VPS33B	1,265279579
HSPA1A	1,257069650
CAP2	1,252439224
GINS4	1,246957448
WDR76	1,242977287
TM7SF2	1,234699620
SAC3D1	1,224673156
DPH6	1,222643861
HSPA1B	1,215876180
MOV10	1,213596632
UAP1L1	1,209023493
SLC11A2	1,207227869
SPR	1,200313081

Table 8B: The highest up-regulated genes in mut*DFNA5* transfected HEK293T cells based on the significant yeast up-regulated genes. Cut-off of fold change was set at 1.2.

Gene symbol	FC_absolute
ZFP36	1,597137261
ACSL4	1,395307021
HDAC4	1,324976457
TCERG1	1,302356201
IQGAP1	1,294371701
DDX3X	1,281575927
USP49	1,261872277
KAT6A	1,242834398
MOB1B	1,235676924
HSPA4	1,231974386
HIPK2	1,231907802
ZFP36L1	1,225043332
KIFC3	1,224887297
CHKA	1,218505094
YTHDF3	1,214666317
